# Barriers to generic antiseizure medication use: Results of a global survey by the International League Against Epilepsy Generic Substitution Task Force

**DOI:** 10.1002/epi4.12583

**Published:** 2022-02-18

**Authors:** Jenna Niyongere, Timothy E. Welty, Michelle W. Bell, Damian Consalvo, Charles Hammond, Howan Leung, Philip N. Patsalos, Melody Ryan, Thanarat Suansanae, Dong Zhou, Hazel Zuellig

**Affiliations:** ^1^ 14314 MercyOne Des Moines Iowa USA; ^2^ College of Pharmacy and Health Sciences Drake University Des Moines Iowa USA; ^3^ Columbia University Irving Medical Center Columbia University New York City New York USA; ^4^ Epilepsy Section Ramos Mejía Hospital Institute of Neurology and Neurosurgery University of Buenos Aires Sanatorio de Los Arcos Buenos Aires Argentina; ^5^ Komfo Anokye Teaching Hospital Kwame Nkrumah University of Science and Technology Kumasi Ghana; ^6^ The Chinese University of Hong Kong Hong Kong China; ^7^ 61554 Department of Clinical and Experimental Epilepsy UCL Queen Square Institute of Neurology London England; ^8^ College of Pharmacy University of Kentucky Lexington Kentucky USA; ^9^ Department of Pharmacy Faculty of Pharmacy Mahidol University Bangkok Thailand; ^10^ West China Hospital Chengdu China; ^11^ Sichuan University Chengdu China; ^12^ Department of Neurosciences College of Medicine San Beda University Manila Philippines; ^13^ 54725 College of Public Health University of the Philippines Manila Manila Philippines; ^14^ 54725 Cardinal Santos Medical Center Metro Manila Philippines

**Keywords:** antiseizure medications, epilepsy, generic medications, generic substitution

## Abstract

The objective of this study was to identify and quantify barriers to generic substitution of antiseizure medications (ASM). A questionnaire on generic ASM substitution was developed by the International League Against Epilepsy (ILAE) Task Force on Generic Substitution. Questions addressed understanding of bioequivalence, standards for generic products, experiences with substitution, and demographic data. The survey was web‐based and distributed to ILAE chapters, their membership, and professional colleagues of task force members. Comparisons in responses were between ILAE regions and country income classification. A total of 800 individuals responded, with 44.2% being from the Asia‐Oceania ILAE Region and 38.6% from European Region. The majority of respondents had little or no education in generic substitution or bioequivalence. Many respondents indicated lack of understanding aspects of generic substitution. Common barriers to generic substitution included limited access, poor or inconsistent quality, too expensive, or lack of regulatory control. Increase in seizures was the most common reported adverse outcome of substitution. Of medications on the World Health Organization Essential Medication list, problems with generic products were most frequent with carbamazepine, lamotrigine, and valproic acid. Several barriers with generic substitution of ASM revolved around mistrust of regulatory control and quality of generic ASM. Lack of education on generic substitution is also a concern. Generic ASM products may be the only option in some parts of the world and efforts should address these issues. Efforts to address these barriers should improve access to medications in all parts of the world.

**Key points**

- Respondents from high‐income countries were more accepting of generic substitution.
- Concerns with generic substitution revolve around regulatory and reliability issues.
- Education of healthcare professionals on generic substitution is important.

## 1 INTRODUCTION

Generic antiseizure medications (ASMs) are often more affordable treatment options for patients. However, there is controversy surrounding generic substitution of an ASM. A systematic review of general attitudes toward generic substitution of any medication, not just ASM, demonstrated that nearly 25% of physicians and pharmacists thought generic medications have increased safety risks and are less effective compared with branded drugs.^1^ Lack of knowledge among healthcare providers about generic medications can increase concerns about the safety of these medications for both healthcare providers and patients. A Nigerian study of opinions on any generic substitution showed approximately 50% of physicians were aware that generic medications can be substituted for originator products, but 72% of these physicians had limited knowledge of bioequivalence and generic substitution principles.^2^ There are no published data focused solely on generic substitution of ASM.

Official policy statements from various International League Against Epilepsy (ILAE) country chapters can create confusion. Some official policies are opposed to generic substitution or only recommend substitution in newly diagnosed people with epilepsy.^3^, ^4^ Other policies support generic substitution only in countries where there are strong regulatory controls and other statements endorse unrestricted substitution.^5^, ^6^ Differing opinions are influenced by a number of factors, such as cultural perspectives, country of practice, and varying interpretations of available literature, and highlight the diverse approaches to use generic seizure medications.

Usually, generic medications must demonstrate bioequivalence to brand products using well‐documented standards. The United States Food and Drug Administration (FDA) Code of Federal Regulations defines bioequivalence as “the absence of a significant difference in the rate and extent to which the active ingredient or active moiety in pharmaceutical equivalents or pharmaceutical alternatives becomes available at the site of drug action when administered at the same molar dose under similar conditions in an appropriately designed study.”^7^ The European Medicines Agency (EMA) has similar but slightly different standards.^8^ Likewise, countries like Russia and China have standards for bioequivalence. Questions about the applicability of these definitions and related standards to patients with epilepsy resulted in multiple prospective and retrospective studies.^9^, ^10^, ^11^, ^12^, ^13^, ^14^, ^15^, ^16^, ^17^, ^18^, ^19^ Retrospective studies had mixed results, but prospective studies showed generic substitution practices to be safe and effective.^9^, ^10^, ^11^, ^12^, ^13^, ^14^, ^15^, ^16^, ^17^, ^18^, ^19^ Most studies were done in high‐income countries with strong regulatory controls, so generalizability to other settings could be questioned.

Ongoing concerns of generic ASM substitution and questions about the applicability of the findings to low‐ and middle‐income countries resulted in the formation of a task force on generic substitution by the ILAE. Task force members created a survey for healthcare providers about generic substitution concerns, to be administered globally. The purpose of this study was to identify concerns and barriers to generic substitution worldwide and determine possible differences between regions and countries.

## 2 METHODS

This study was approved by the Drake University Institutional Review Board (approval #2018‐19100). A survey of 26 questions was developed by the ILAE Task Force on Generic Substitution, using a modified Delphi Method. The survey was not formally validated. However, after establishing the question set, the survey was independently reviewed by faculty, with expertise in survey development, administration, and analysis, in the College of Business and Public Administration (CBPA) at Drake University. The CBPA faculty provided input into question edits, sequencing of questions, and survey distribution that were incorporated into the project. Survey questions assessed perceptions of generic medications related to safety, efficacy, regulations, and knowledge of generic medications.

The survey was available to access online from April 2019 to February 2020. A Google Translate option was included in the survey to allow respondents to have translation into most major languages. The survey was administered online using a survey platform (Qualtrics). The full survey is available in Appendix S1. A link to the survey and explanation of the survey was sent to ILAE chapters for distribution to their individual members. The method of distribution to individual members varied between chapters to include an announcement in regular newsletters to personal contact with individual members through email. Participation in distributing the survey information by the chapters was entirely voluntary. Additionally, task force members were encouraged to provide information on the survey to professional colleagues and contacts. When the survey was accessed, participants were informed about the purpose of the study prior to starting the survey and given an option to opt out of the survey. Survey questions could be skipped, if the respondent desired. Formal follow‐up email reminders were sent twice within 3 months of the original email. Additionally, task force members reminded colleagues of the survey.

The World Bank classification of countries according to Gross National Income was used to categorize countries as low‐, middle‐, and high‐income and compare results.^20^ Descriptive statistics were used to display results. While data on all seizure medications were collected, we only analyzed data on medications in the World Health Organization Essential Medication list.^21^ We also analyzed data in relationship to the ILAE region where the respondent practiced.^22^

## 3 RESULTS

A total of 800 individuals from all ILAE regions responded to the survey. Respondents were predominantly from Asia‐Oceania (n = 261) and Europe (n = 228). The majority of responses (Table 1) were physicians (n = 546, 92.1%). Most respondents practiced in a university hospital (n = 185, 31.3%) or a government public hospital (n = 185, 21.6%).

**TABLE 1:** Summary of answers to survey questions

Survey Question (Numbers correspond to the question numbers on actual survey.)	Options	Responses (%)
1. Prescriptive practices (n = 737)	Must have prescription	658 (89.3)
	No prescription needed	13 (1.8)
	Prescription for controlled substance only	66 (9.0)
2. Level of regulatory control (n = 718)	Extensive	397 (55.3)
	Moderate	273 (38.0)
	Poor	48 (6.7)
3. Amount of education on generic substitution (n = 706)	None	189 (26.8)
	One continuing education program	112 (15.7)
	More than one continuing education program	268 (38.0)
	Research on generic medications	64 (9.1)
	Other	73 (10.3)
4. Drug products available in your country (n = 702)	Only brand name (no generics)	17 (2.4)
	Only approved generic products (no brand names)	7 (1.0)
	Brand name and approved generic products	576 (82.1)
	Brand name, approved, and unapproved generic products	93 (13.3)
	Only approved and unapproved generic products (no brand name)	0 (0)
	Only products on essential medication list	1 (0.1)
	Other	8 (1.1)
5. Definition of Bioequivalence (n = 691)	Drug products that are therapeutic equivalents for the same indication	387 (56.0)
	Drug products with equivalent absorption of the same drug (correct answer)	275 (39.8)
	Different dosage forms of the same drug	8 (1.2)
	I do not know	21 (3.0)
6. Standards for bioequivalence established by (687)	National or federal government	325 (47.3)
	Provincial, state, or local government	10 (1.5)
	Standards from other countries adopted	85 (12.4)
	Pharmaceutical companies	82 (11.9)
	I do not know	165 (24.0)
	Other	20 (2.9)
7. Adopt standards from other countries (n = 650)	EMA	194 (29.6)
	USFDA	201 (30.9)
	HealthCanada	9 (1.4)
	PharmRussia	1 (0.2)
	I do not know	197 (30.3)
	Other	48 (7.4)
9. Believe that generic products equally safe and effective (n = 688)	Yes	269 (40.3)
	No	151 (22.6)
	Undecided	248 (37.1)
10. Greatest concern with generic products (n = 785)	Limited access to generic products	92 (11.7)
	Poor or inconsistent quality	286 (36.4)
	Too expensive	53 (6.8)
	Lack of regulatory control	202 (25.7)
	Other	152 (19.4)
11. Percent of patients you prescribe for AED (n = 641)	Less than 10%	62 (9.7)
	11%‐25%	147 (22.9)
	26%‐50%	132 (20.6)
	51%‐75%	134 (20.9)
	More than 75%	166 (25.9)
12. Patients automatically switched to generic product (n = 654)	Always	30 (4.6)
	Always, unless physician or prescriber designates dispense as written	227 (34.7)
	No	352 (53.8)
	Unsure	45 (6.9)
13. Percent of prescriptions you write for generic products (n = 638)	Less than 10%	117 (18.3)
	11%‐25%	100 (15.7)
	26%‐50%	185 (29.0)
	51%‐75%	110 (17.2)
	More than 75%	126 (19.8)
14. Percent of patients actually taking generic product (n = 641)	Less than 10%	70 (10.9)
	11%‐25%	102 (15.9)
	26%‐50%	205 (32.0)
	51%‐75%	166 (25.9)
	More than 75%	98 (15.3)
15. Percent of patients who actually receive the product written on the prescription (n = 635)	Less than 10%	76 (12.0)
	10%‐25%	68 (10.7)
	26%‐50%	102 (16.1)
	51%‐75%	74 (11.7)
	More than 75%	259 (40.8)
	I do not know	56 (8.8)
16. What have you observed with generic substitution (n = 609)	Increase seizure frequency	245 (40.2)
	Change in seizure semiology or characteristics	40 (6.8)
	Increased dose‐ or concentration‐related toxicity	105 (17.2)
	Increased allergic or idiosyncratic toxicity	55 (9.0)
	Other	164 (26.9)
19. Most agree with this statement (n = 593)	All approved generic products should be considered therapeutically equivalent to brand products	210 (35.4)
	Some approved generic products should be considered therapeutically equivalent to brand products	253 (42.9)
	No approved generic product should be considered therapeutically equivalent to brand products	28 (4.7)
	I need more information about substitution of a generic for a brand product	102 (17.2)
20. Profession (n = 596)	Medical doctor	549 (92.1)
	Nurse	11 (1.9)
	Nurse practitioner/advanced nurse practitioner	16 (2.7)
	Pharmacist	8 (1.3)
	Physician assistant	2 (0.3)
	Clinical officer	1 (0.2)
	Psychologist	0 (0)
	Other	9 (1.5)
21. Specialty (n = 594)	Primary care physician	8 (1.4)
	General adult neurologist	190 (32.0)
	General child or pediatric neurologist	89 (15.0)
	Adult epileptologist	154 (25.9)
	Child or pediatric epileptologist	65 (10.9)
	Neurosurgeon	13 (2.2)
	Psychiatrist	14 (2.4)
	Psychologist	0 (0)
	Neurodevelopmental pediatric specialist	7 (1.2)
	General nurse	0 (0)
	Primary care nurse practitioner	0 (0)
	Epilepsy nurse practitioner	25 (4.2)
	General pharmacist	3 (0.5)
	Board certified pharmacy specialist	3 (0.5)
	Physician assistant	0 (0)
	Pediatrician	7 (1.2)
	Other	15 (2.7)
27. Practice setting (n = 592)	Community Private Hospital	66 (11.5)
	University Hospital	185 (31.3)
	Government Public Hospital	128 (21.6)
	Primary Care Clinic	14 (2.4)
	Secondary Care Clinic	12 (2.0)
	Tertiary Care Clinic	47 (7.9)
	Neurology Clinic	62 (10.5)
	Specialty Epilepsy Center	46 (7.8)
	Other	32 (5.4)
22. Age (n = 593)	Less than 29 years	31 (5.2)
	30‐39 years	133 (22.4)
	40‐49 years	173 (29.2)
	50‐59 years	147 (24.8)
	60‐69 years	88 (14.8)
	70 years or older	21 (3.5)
23. Sex (n = 592)	Female	330 (55.7)
	Male	262 (44.3)
24. ILAE region of practice (n = 591)	Africa	28 (4.7)
	Asia‐Oceania	261 (44.2)
	North Africa and Eastern Mediterranean	12 (2.0)
	Europe	228 (38.6)
	Latin America	46 (7.8)
	North America	16 (2.7)

Nearly 90% of respondents indicated that prescriptions were required for patients to obtain seizure medications. Eighty‐two percent stated that brand name and approved generic products were available in their country, and another 13% responded that brand name, approved generic products, and unapproved generic products were available. Over half of respondents stated that automatic switches between brand and generic products were not allowed in their country.

Forty percent of respondents indicated they observed increased seizure frequency they related to generic substitution. Increased adverse medication effects related to generic substitution were reported by approximately 17% of respondents. Most respondents thought the products that were prescribed were the products patients were actually taking.

**FIGURE 1:**
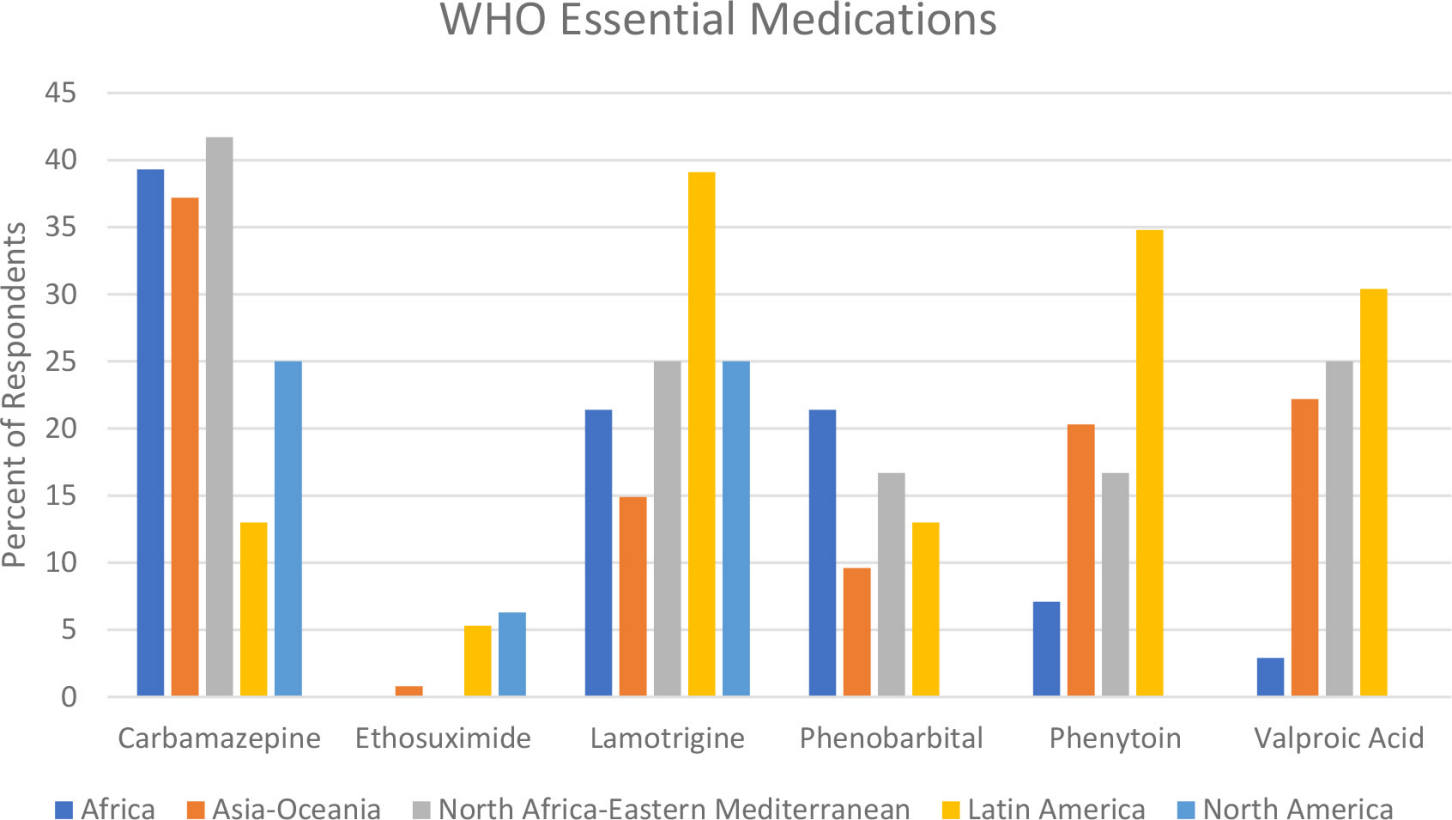
Percent of respondents from various ILAE regions who reported problems with generic antiseizure medications (ASM) products from the list of WHO essential medications

Differences in regulatory control were seen between respondents from high‐, middle‐, and low‐income countries (Figure 1). Seventy nine percent of respondents in high‐income countries noted extensive regulatory control, while approximately 38% in middle‐income and 35% in low‐income countries indicated extensive regulatory control. Inconsistent quality of drug products was of primary concern to all respondents, regardless of where they were located.

A larger proportion of individuals from high‐income regions felt that generic medications are equally safe and effective as brand name drugs, when compared to low‐ and middle‐income regions. When asked to comment on their selection of ‘yes’ or ‘no’ of whether they felt generic ASMs are as safe and effective as branded drugs, many responded ‘no’ due to concerns of quality of generics or responded ‘yes’ on the condition that the generic is of good quality. The responses were grouped based on the income level of the country that the individual indicated they resided. In high‐income countries, 46.5% of respondents answered ‘yes’ as compared to 26.7% in middle‐income and 35.9% in low‐income countries.

**FIGURE 2:**
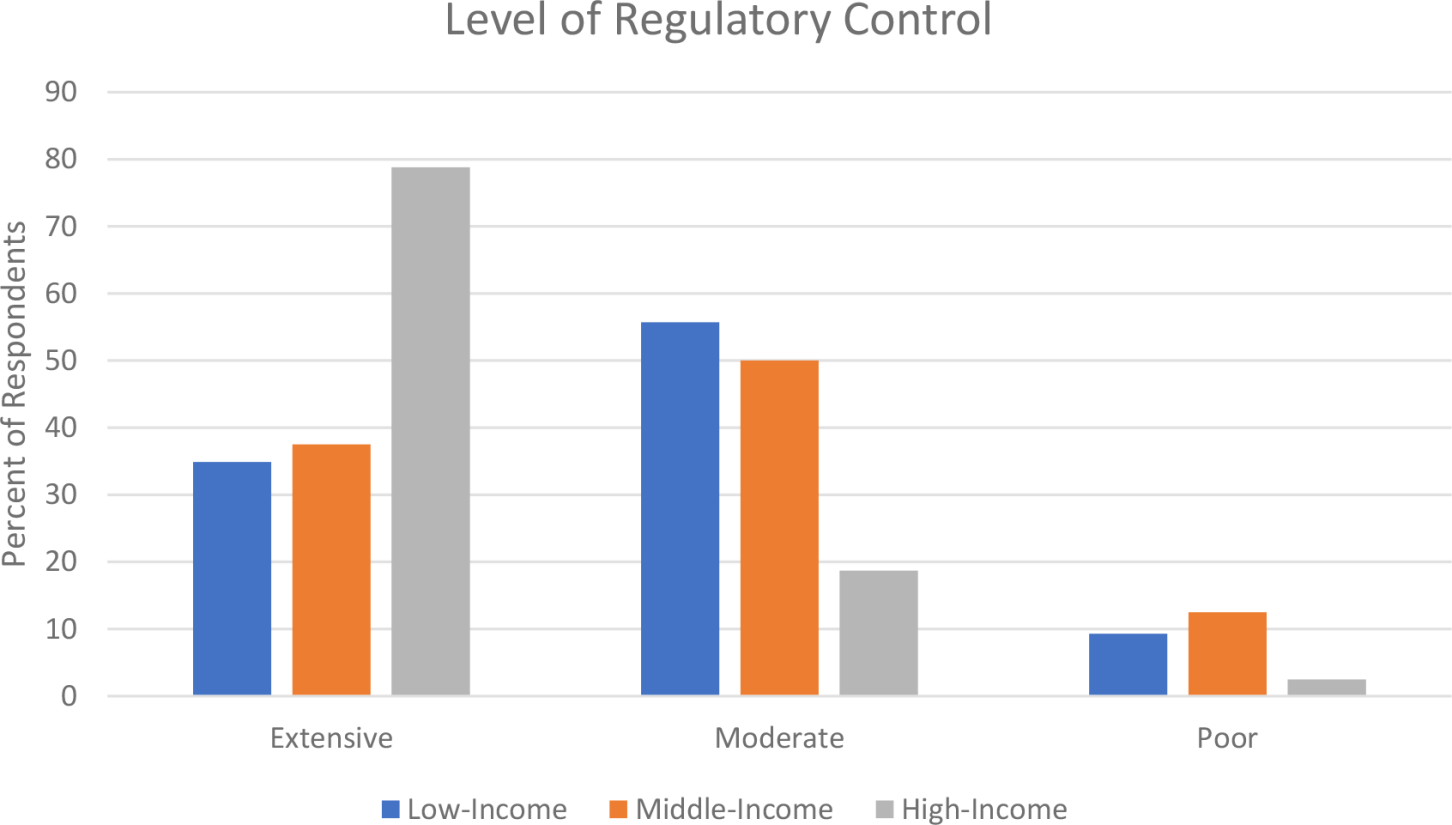
The percent of respondents who reported extensive, moderate, or poor regulatory control of pharmaceutical products in their country

Twenty‐five percent or more of respondents reported problems with generic carbamazepine, lamotrigine, and valproic acid (Table 2), all of which are on the WHO Essential Medications List. The ILAE Latin American region more consistently reported problems with generic products across the various seizure medications than the other ILAE regions (Figure 2).

**TABLE 2:** Availability of generic products and reported problems

Seizure medication	Generic available (n)	Problem with generic encountered (n, %)
Acetazolamide	412	17, 12.0
Brivaracetam	185	11, 5.9
Cannabidiol	116	18, 15.5
Carbamazepine^a^	551	224, 40.7
Clobazam	322	33, 10.2
Diazepam^a^	524	37, 7.1
Divalproex sodium	370	95, 25.7
Eslicarbazepine	161	10, 6.2
Ethosuximide^a^	283	16, 5.6
Felbamate	141	7, 5.0
Gabapentin	529	53, 10.0
Lacosamide	412	18, 4.4
Lamotrigine^a^	535	159, 29.7
Levetiracetam	545	194, 35.6
Lorazepam^a^	293	14, 4.8
Oxcarbazepine	483	74, 15.3
Perampanel	340	11, 3.2
Phenobarbital^a^	520	52, 10.0
Phenytoin^a^	486	90, 18.5
Primidone	231	7, 3.0
Rufinamide	191	7, 3.7
Stiripentol	160	6, 3.8
Topiramate	526	97, 18.4
Valproic acid^a^	540	134, 24.8
Zonisamide	355	14, 3.9
Other	37	10, 27

^a^ WHO essential medication.

Nearly half of respondents in high‐ and middle‐income countries reported no or minimal education on bioequivalence and generic substitution, compared to a third of respondents from low‐income countries (Table 3). In low‐income countries, over half of respondents had attended more than one education program on generic medications. High‐income countries had lower rates of participating in an educational program but higher rate of engaging in research on generic substitution. More than half of the respondents had difficulty identifying the correct definition of bioequivalence (Table 1).

**TABLE 3:** Amount of education on generic medications and country income level

Income level	No education or training (%)	Attended one continuing education program (%)	Attended >than one continuing education program (%)	Participated in research (%)	Other (%)
Low (n = 287)	14.3	17.4	54.7	4.2	9.4
Middle (n = 58)	36.2	12.1	31.0	6.9	13.8
High (n = 242)	35.1	13.6	25.2	16.1	9.9

Over 75% of respondents did agree with statements that all or some generic products are therapeutically equivalent to the brand product (Table 1). Nearly 20% of respondents indicated they needed more information on generic substitution to determine whether they agree that all or some generic products are equivalent to brand products.

## 4 DISCUSSION

The objective of this study was to identify and quantitate barriers to generic ASM substitution through a survey sent to healthcare providers worldwide. We received 800 responses from providers, the largest survey of its type. While the survey was widely distributed, responses were limited to individuals with Internet access, potentially missing healthcare providers in rural and remote areas of the world.

One objective of the analysis was to determine whether country income level influenced the healthcare providers' identification of barriers to generic substitution. When results were divided by country income level, more respondents from high‐income countries felt generic medications are as safe and effective as brand name compared with middle‐ and low‐income countries. The precise reasons for this finding were unclear, but poor regulatory control of generic medications and inconsistent quality of generic products were the biggest concerns among respondents from low‐income countries. As noted in the survey results, respondents from low‐income countries noted fewer regulatory controls. This is complicated by the fact that there is no single international standard for the determination of bioequivalence, and approval of generic products and standards vary widely between countries.^23^, ^24^ Some professional pharmaceutical science organizations and the WHO have noted this to be a problem and have recommended international harmonization of definitions and standards for bioequivalence and generic product approval.^25^, ^26^, ^27^, ^28^ Many high‐income countries, like the United States and several European countries, have strong regulatory controls, policies, and monitoring, which prevent poor quality or unproven generic products from being marketed. Medication dispensing practices, cultural factors, and health beliefs could also be factors in the lack of confidence in generic substitution among individuals in low‐income countries. Further investigation into reasons for less confidence in generic products among respondents from low‐income countries is needed.

The survey did not capture data on increased use of more expensive branded products due to concerns with generic ASM products. Overall costs of care could increase by shifting of prescribing patterns in low‐income countries to more use of branded ASMs. Likewise, increased regulation of generic ASM products could increase their cost, due to the expense of adhering to more rigorous regulations, but improve product quality and consistency. Increased regulatory‐related costs might offset some or all of the cost advantage associated with the use of generic ASM products. This is an area that needs to be more thoroughly explored.

Education and training about generic substitution is also a concern. Individuals in low‐income countries reported attending continuing education on generic substitution more frequently than those in higher income countries (Table 3). This is an interesting finding, given the fact that individuals from low‐income countries had less confidence in generic ASM substitution than those in high‐income countries. It is unclear why this was true, but confusion about the definition of bioequivalence seen in this study seems to indicate that additional unbiased education in general is needed. To improve generic ASM substitution, practitioners should be provided a higher quantity and more thorough unbiased education about generic ASMs, their production, approval process, and bioequivalence standards. A basic understanding of bioequivalence and generic drug products is essential to appropriate substitution of ASMs in people with epilepsy. These results indicate where targeted unbiased education programs can be helpful in improving the use of generic products.

Important to note is that 3 ASMs on the WHO Essential Medications list, carbamazepine, valproic acid and lamotrigine, were most frequently perceived to have problems with generic substitution. It is unclear why these ASMs had a high frequency of complaints. These are commonly used ASMs, so wide use may have resulted in at least the perception of more problems with generic substitution. This theory may be substantiated by the low rate of problems noted with ethosuximide, an ASM with a very narrow spectrum of activity and less use. Based on the pharmaceutical properties of carbamazepine and lamotrigine (eg, poorly water soluble), it is conceivable that generic products could be inconsistent in quality.^29^ However, the pharmaceutical properties of valproic acid (eg, highly water soluble) make this explanation less likely.^29^ Formal tracking on a global basis of reported problems with various generic products could be helpful in identifying the true frequency of these reports and specific generic products that are problematic.

Problems reported with generic products did not favor one ILAE region over another. However, it appears that higher percentages of respondents from Africa, Asia‐Oceania, North Africa‐Eastern Mediterranean, and Latin America reported problems with various generic ASMs. These regions contain the highest percentages of low‐ and middle‐income countries, raising the possibility that lower quality or less consistent ASM products are dispensed in these countries. Certainly, respondents from these countries were more likely to report moderate or poor regulatory control of medications. More research needs to be done to consider the connection between regulatory controls and reports of problems with generic ASM products.

Third‐party payors, such as insurance companies and government health programs, also play a large role in generic substitution. Restrictive formularies, policies requiring generic substitution, and step‐therapy requirements impact selection of medications and substitution of drug products. We did not assess the role of third‐party payors and provider understanding of the impact these entities have on generic substitution. Future studies should take this factor into account when evaluating generic substitution practices.

Most respondents were at least partially accepting generic substitution and willing to consider that generic products are equivalent to brand products. However, a good percentage of respondents indicated they need more information about generic substitution before rendering a decision on the legitimacy of substituting generic products for branded products. Additional education and general guidance on generic substitution may be helpful in addressing the concerns of individuals who are hesitant to endorse this practice. Education will also raise awareness and the ability to appropriately manage generic ASM substitution in countries where supplies of products may be regularly switched due to the disruption of consistent supply chains of individual products.

While there was good correlation in our survey between what product respondents said they prescribed and what products they thought their patients were taking, this may not be an entirely accurate representation of what is occurring. Previous studies have demonstrated that physicians underestimate the use of generic ASM products among their patients.^30^ Practices like forced substitution, unless the prescriber indicates that a particular product is medically necessary, result in generic substitution even when a prescription is written for a brand ASM product, and prescribers may be unaware of the substitution. It would be useful to globally survey or evaluate what ASM product patients actually receive compared to what was prescribed.

The distribution of respondents from countries within the various ILAE regions was not balanced in several ways. First, the majority of respondents were from the European and Asia‐Oceania regions, with few respondents from the other regions. Additionally, the large majority of respondents in the European region were from Italy and in the Asia‐Oceania region were from the Philippines (ie, over 50% of respondents in these regions were from these countries). We attempted to address this problem by keeping the survey open longer than planned, sending multiple reminders to complete the survey, and contacting colleagues in these regions with information on the survey. Reasons for the disproportionate responses from various regions could include smaller total numbers of ILAE members or neurologists in some of the poorly represented regions or perceptions that generic substitution in not a concern. These discrepancies could have skewed our results to make them more representative of an European or Filipino perspective. However, we attempted to attenuate this influence by including in our analysis the income designation of countries of residence for respondents. Other limitations to the survey include that it was administered in English, and that the survey may not have been accessible to individuals living in locations with limited Internet access. A Google translate option was available to non‐English‐speaking respondents, but the translation that was rendered may not have accurately conveyed the meaning of individual questions. Given these limitations with our survey, our results are remarkably similar to findings in a systematic review of international literature on physician and pharmacist views of generic substitution.^31^

In conclusion, we identified that the perception of limited regulatory control and inconsistent product quality, and little education on bioequivalence and generic substitution were major barriers to the use of generic ASM products.

## CONFLICT OF INTEREST

Dr Niyongere has no conflicts to disclose. Dr Welty has no conflicts to disclose. Dr Bell has no conflicts to disclose. Dr Consalvo has no conflicts to disclose. Dr Hammond has no conflicts to disclose. Dr Leung discloses that he is the President of the Hong Kong Epilepsy Society and serves on the drug utilization committee of the Prince of Wales Hospital in Hong Kong. Dr Patsalos has no conflicts to disclose. Dr Ryan has no conflicts to disclose. Dr Suansanae has no conflicts to disclose. Dr Zhou discloses that he is the deputy editor of Epilepsia Open. Dr Zuellig has no conflicts to disclose. We confirm that we have read the Journal's position on issues involved in ethical publication and affirm that this report is consistent with those guidelines.

## DISCLAIMER

This report was written by experts selected by the International League Against Epilepsy (ILAE) and was approved for publication by the ILAE. Opinions expressed by the authors, however, do not necessarily represent the policy or position of the ILAE.

## Supporting information

Appendix S1
