# Supplementary material for: Barriers to generic antiseizure medication use: Results of a global survey by the International League Against Epilepsy Generic Substitution Task Force
Source: Epilepsia Open. 2022 Feb 18;7(2):260–70. doi: 10.1002/epi4.12583 (PMC9159248; doi:10.1002/epi4.12583)
Supplement: Supplementary file 1 — Appendix S1 [file EPI4-7-260-s001.docx]

Appendix 1

Complete Survey

Generic Drug Survey

You are invited to participate in a study survey from the Generic Substitution Task Force of the International League Against Epilepsy (ILAE). The purpose of this survey is to better understand the knowledge, practices, and policies worldwide related to generic substitution of medications for epilepsy. Information from this survey will be used to guide educational programs from the ILAE, direct policy statements, and identify barriers to generic substitution of medications for epilepsy. Data from this survey will be analyzed in aggregate and may be presented at professional meetings or published in professional journals. All responses will be anonymous. The Institutional Review Board of Drake University in Des Moines, Iowa, USA has approved this study. Information about the approval of this study can be directed to the IRB Administrator at irb@drake.edu or 1-515-271-3472. Specific questions or concerns regarding this study can be directed to the Principal Investigator, Timothy Welty PharmD, at timothy.welty@drake.edu or 1-515-271-2762. Please complete all the survey questions to the best of your ability. If you are uncomfortable answering a specific question, you can decide not to respond that item and continue through the survey. Thank you in advance for your participation in this study.

o I consent

o I do not consent

Which of the following statements best describes practices in your country?

o Patients must have a prescription from a physician or other healthcare provider to obtain antiepileptic (antiseizure) medicine.

o Patients do not need a prescription from a physician or other healthcare provider to obtain antiepileptic (antiseizure) medicine.

o Patients are only required to have a prescription from a physician for controlled or restricted use antiepileptic (antiseizure) medicine (e.g., barbiturates, benzodiazepines). Other antiepileptic (antiseizure) medicines do not require a prescription.

What is the level or regulatory control over the production of pharmaceutical products in your country?

o Extensive (e.g., strong standards and are regularly enforced)

o Moderate (e.g., less strong standards, or standards are inconsistently enforced)

o Poor (e.g., weak or no standards, or standards are not enforced)

What education or training on generic medications in epilepsy have you received?

o No education or training

o Attended one continuing education program on generic medications

o Attended more than one education program on generic medications

o Participated in research studies of generic medications

o other ________________________________________________

What drug products of antiepileptic drugs are available in your country?

o Only brand name drug products (no generic products)

o Only approved generic products (No brand name products)

o Brand name and approved generic products

o Brand name, approved and unapproved generic products

o Only approved and unapproved generic products (no brand name products)

o Only products on an essential medication list

o other (7) ________________________________________________

What does the term bioequivalence refer to?

o Drug products that are therapeutic equivalents for the same indication.

o Drug products with equivalent absorption of the same drug.

o Different dosage forms (e.g., extended release) of the same drug.

o I do not know

In your country, standards for bioequivalence are established by which of the following?

o The national or federal government of your country.

o The provincial, state, or local governments of your country.

o Standards from other countries are adopted.

o Pharmaceutical companies

o I do not know

o other ________________________________________________

If bioequivalence standards from other countries are adopted, what standards does your country use?

o European Medicines Agency

o United States Food and Drug Administration

o Health Canada

o PharmRussia

o I do not know

o other ________________________________________________

Do you believe that generic drug products available in your country are equally safe and effective to brand name drug products? Please comment on your selection

o Yes ________________________________________________

o No ________________________________________________

o Somewhat ________________________________________________

What is your greatest concern with prescribing generic antiepileptic (antiseizure) drugs available in your country? (Select all that apply)

▢ Limited access to generic drug products

▢ Poor or inconsistent quality of generic drug products

▢ Generic drug products are too expensive

▢ Lack of regulatory control over generic drug products

▢ Other ________________________________________________

In your daily practice, what is the percentage of patients that you prescribe AEDs?

o Less than 10%

o 11 to 25%

o 26 to 50%

o 51 to 75%

o More than 75%

In your country, are your patients automatically switched to a generic product?

o Always

o Always, unless physician or prescriber designates “dispense as written”

o No

o Unsure

What percentage of prescriptions you write are written for generic drugs?

o Less than 10%

o 11-25%

o 26-50%

o 51-75%

o More than 75%

What percentage of your patients are actually taking generic products?

o Less than 10%

o 11-25%

o 26-50%

o 51-75%

o More than 75%

What percent of the prescriptions your patients receive are filled with the specific product name (e.g., brand name, specific generic name) product written on the prescription?

o 0-10%

o 11-25%

o 26-50%

o 51-75%

o More than 75%

o I do not know

Based on your experience, which of the following do you believe you have actually observed in one of your patients taking a generic medication for seizures?

o Increased seizure frequency

o Change in seizure semiology or characteristics

o Increased dose or concentration-related toxicity (e.g., sedation, drowsiness, ataxia, diplopia, dizziness)

o Increased allergic or idiosyncratic toxicity (e.g., rash, angioedema, neutropenia, hepatotoxicity)

o Other ________________________________________________

Please answer the following questions regarding availability of generic medications and problems you have experienced with generic medications. (Check all answers in both columns that apply.)

Which antiepileptic (antiseizure) medications are available in your country? Which of the following antiepileptic (antiseizure) medications have you experienced problems with generic products?

Answer 1 Answer 1
